# Resilience, trauma, and hopelessness: protective or triggering factor for the development of psychopathology among migrants?

**DOI:** 10.1186/s12888-020-02729-3

**Published:** 2020-07-08

**Authors:** Eleonora Gambaro, Martina Mastrangelo, Marco Sarchiapone, Debora Marangon, Carla Gramaglia, Camilla Vecchi, Chiara Airoldi, Concetta Mirisola, Gianfranco Costanzo, Silvia Bartollino, Francesca Baralla, Patrizia Zeppegno

**Affiliations:** 1 Department of Translational Medicine, Institute of Psychiatry, University of Western Piedmont, via Solaroli, 17, 28100 Novara, Italy; 2 Psychiatric Ward, Maggiore della Carità University Hospital, Corso Mazzini 18, 28100 Novara, Italy; 3 Department of Medicine and Health Science, University of Molise, Via De Santis, 86100 Campobasso, Italy; 4 National Institute of Migration and Poverty, Via San Gallicano 25, Rome, Italy; 5 National Medical University, Almaty, Kazakhstan

**Keywords:** Resilience, Trauma, Hopelessness, Migration

## Abstract

**Background:**

Recently, many studies have investigated the role of migration on mental health. Nonetheless, only few focused on the consequences of childhood trauma, hopelessness, and resilience on migrants’ psychopathology, including psychiatric disorders and symptoms.

**Method:**

119 migrants were recruited between May 2017 and April 2018, among those applying for assessment to the Mental Health Operational Unit of the National Institute for Health, Migration and Poverty (NIHMP) in Rome, Italy. Assessment included the following: Zung Self-Rating Depression Scale (SDS), Zung Self-Rating Anxiety Scale (SAS), Connor-Davidson Resilience Scale (CD-RISC), Childhood Trauma Questionnaire (CTQ), Posttraumatic Stress Disorder Checklist for DSM-5 (PCL-5), Beck Hopelessness Scale (BHS), Beck’s Suicide Intent Scale (SIS), Brief Aggression Questionnaire (BAQ), Deliberate Self-Harm Inventory (DSHI).

**Results:**

53.39% of migrants scored above the PCL-5 cut-off score (mean score was 39.45). SDS scores below the cutoff suggested the presence of depression in 42.37%, while According to SAS scores anxiety levels were low in 38.98% of migrants. During childhood, physical abuse and neglect were reported respectively by 56.78 and 69.49% of migrants.

**Conclusion:**

We found that Post Traumatic Stress Disorders play the role of mediators for the relation between the childhood traumatic experiences and aggressiveness, anxious and depressive symptomatology, while hopelessness is a mediator between the childhood traumatic experiences and the development of depression in adulthood. Hopelessness seems to influence the strength of the relation between childhood traumatic experiences and the individual’s current intensity of suicidal attitudes, plans, and behaviors. Further developments and future perspectives of the research project are to address key gaps in the field of resilience by means of a longitudinal evaluation study in migrants, including a native population control group, acceding to NIHMP.

## Background

We live in a world currently marked by demographic changes, where migration is currently, and will be in the future as well, a hot topic. The increasing number of migrants arriving in Europe represents a humanitarian, political and healthcare challenge [1, 2]. Particularly, the adverse impact of migration on mental health is well established, and likely due to multifaceted causes including traumatic events exposure, daily stressors and impoverishment [3], separation from parents and friends, and difficulties adjusting to a new, stranger environment [4]. Lazarus and Folkman in 1984 [5] stated that the *experience of stress and coping becomes more salient when individuals are faced with major life changes or challenges* [6]. These include the migration process, which is generally considered as a major chronic environmental stressor, requiring immense coping skills and abilities to adapt to a new and often adverse environment [7]. Castro and Murray introduced the concept of resilience in the stress-coping model firstly developed by Lazarus and Folkman [8]. Resilience is *the ability to positively cope with adverse situations or the ability to bounce back after experiencing an adverse situation* [9]. Resilience, together with coping strategies, may help individuals in facing adversity [10]. The individual ability to face personal or environmental difficulties, while maintaining positive outcomes, constitutes the risk-and-resilience perspective [11], which may be applied to challenging experiences like migration [12].

Recently, a systematic-narrative review of reviews about mental health in first-generation migrants reported a high level of one or more common mental disorders in this population (i.e. depression, anxiety, post-traumatic stress disorder, and postpartum depression) [13]. In particular, it is well established that the prevalence of depression and depressive symptoms in adult ethnic minorities is significantly higher than in native populations in Europe [14–16]. Nonetheless, depression is underdiagnosed in migrants [17] due to multiple reasons, including the lack of words describing depression in several languages and the fact that in some cultural contexts depression is considered as normal life ups and downs, not needing medical treatment [18].

Albeit receiving growing attention in the depression research field, the construct of hopelessness is still understudied in migrant populations. Hopelessness can be defined as a trans-diagnostic psychological construct characterized by rigid and persistently negative expectations about the future and helplessness to change future outcomes [19]. It is associated with psychosocial risk factors, psychopathology and high-risk behaviors, such as deficit in cognitive development [20], negative inferential style [21], low socio-economic status [22], stress [23], emotional abuse [24], more severe depressive symptoms, suicidal ideation, substance abuse, aggression and violent behaviors [21].

Another key issue typically implicated in the migration process is trauma. Indeed, migrants often live a set of traumatic experiences in their country of origin (i.e. war, tortures), during the migration period, and/or during the resettlement process in the new country [1]. The impact of traumatic experiences on the onset of post-traumatic stress disorder (PTSD) is widely acknowledged; PTSD may develop after a single or prolonged exposure to exceptionally threatening or horrifying events [25]. The overall prevalence of PTSD in migrants was 47% in a meta-analysis conducted on 20 pooled studies published during 1994 and 2007 [26], while the lifetime prevalence of PTSD in the general population usually ranges from 1.9% to 8.8% [25]. Moreover, it has been highlighted that the exposure to more risk factors, such as violence, war, and political persecution may be related to a higher prevalence of PTSD among refugees, such as in labor migrants [27]. More specifically, the literature shows that not only multiple traumatic events, being a victim of violence (e.g., torture, rape/ sexual assault, armed conflicts), and economic hardship, may play the role of risk factors for PTSD, but also post-migration difficulties, as a poor social network, lack of counseling services, socioeconomic/political problems, detention, language barrier and acculturative stress [7].

Linked to traumatic experience, in recent years childhood trauma has gained increasing attention. While a vast body of literature has pointed to a major role of early childhood adversities on the development of psychiatric disorders in adulthood, studies regarding child maltreatment or emotional/behavioral problems among migrants and their possible consequences in adulthood are scarce [28].

As described above, while on the one hand, we have stressors and vulnerability, on the other we may have “protective” factors, whose complex interplay with the risk and vulnerability ones should be carefully assessed. Actually, emerging data over the past decades suggest that migration research could benefit from using a strengths-based approach, including the resilience construct, for a more thorough understanding of migrants’ experiences [29, 30]. Resilience may represent an essential element of epidemiological and interventional research aimed at improving mental health outcomes in migrants [3], because of its impact on how migrants adapt to the migration process and on the acculturation experiences in their host countries [1, 2, 31].

While in recent years a growing number of studies shed light on the role of migration process on mental health, few studies focused their attention on specific aspects of this process. Specifically, data assessing the consequences of childhood trauma, hopelessness, and resilience on migrants’ psychopathology are still controversial. In the attempt to fill this gap, we carried out a study focused

on this understudied topic, in order to allow a better understanding of the phenomenon and the development of targeted clinical interventions and prevention programs.

We hypothesize that childhood trauma correlates with anxiety and depression severity among first generation migrants (Hypothesis =0); we also hypothesize that hopelessness may lead to a further worsening of mental health disorders severity among migrants, whereas resilience, hopelessness and post traumatic stress symptoms may act as a mediating or moderator factors.

## Methods

Approval for the research was obtained from the Comitato Etico Istituto Superiore della Sanità (Prot. PRE/17) and informed consent was obtained from each participant, conforming to the Helsinki Declaration.

### Setting

The National Institute for Health, Migration and Poverty (NIHMP), set in Rome from 2006, is the national referral center for migration and poverty-related social and healthcare issues and for transcultural mediation in the healthcare field. The NIHMP is a chosen place for the reception of disadvantaged people refugees, asylum seekers, homeless people, victims of torture, etc.. The staff working in this center offers help to people in three main situations: a) emergency: primary assistance is provided to migrants in arrival areas, in close collaboration with social and healthcare operators, under the supervision of the Prefectures; b) initial process of social inclusion of newly-arrived migrants; c) run-down city areas, where social exclusions and poverty are more frequently reported.

### Participants

One-hundred and nineteen migrants were recruited (including 102 males and 17 females), in the period between May 2017 and April 2018, among those applying for assessment to the Mental Health Operational Unit of the National Institute for Health, Migration and Poverty (NIHMP) in Rome, Italy.

After the initial interview, migrants were informed about the study design and asked about their willingness to participate in the research, on a voluntary and anonymous basis, after signing an informed written consent. The study received Ethical approval from the Istituto Superiore della Sanità (ISS) (Prot. PRE 292/17, 18^th^ April 2017). Reporting is consistent with all ethical requirements.

### Assessment and measures

The research protocol was built ad hoc with the aim of gathering relevant information about migrants’ socio-demographic and clinical features, previous and current psychiatric diagnosis (categorized for the statistical analyses in psychotic, neurotic and traumatic disorders), suicidal behaviors and suicide attempts.

The face-to-face interview was performed by trained interviewers, in a single session. The interview consists of multiple modules covering demographic characteristics (e.g., age, sex, marital status, education/training, employment, migrant status and self-reported socioeconomic status), lifestyles and behaviors, psychiatric symptoms, current and previous psychiatric diagnosis, family history of psychiatric disorders, individual and family history of suicide and suicidal behavior, childhood and adulthood trauma history.

### Measures

Patients were asked to complete the following self-administered questionnaires and scales, translated and validated in English and Italian languages:

- Zung Self-Rating Depression Scale (SDS) [32]

Short self-administered 20-item survey rating four common characteristics of depression: pervasive affect, physiological equivalents, other disturbances, and psychomotor activities. Each question is scored on a scale ranging from 1 to 4 points, hence overall scores range from 25-100, with the following cutoff values: 25-49 normal range; 50-59 mild depression; 60-69 moderate depression; 70 and above severe depression.

- Zung Self-Rating Anxiety Scale (SAS) [33]

This self-administered scale is focused on the most common general anxiety disorders. Each response is rated on a 4-point scale, from ‘none of the time” to “most of the time.” There are 20 questions with 15 increasing anxiety level questions and 5 decreasing anxiety questions. There are two formats, self-evaluations and clinical evaluations [34].

- Connor-Davidson Resilience Scale (CD-RISC) [31]

This is a new rating scale for the assessment of resilience, comprising 25 items evaluated on a five-point Likert scale ranging from 0-4: not true at all (0), rarely true [1], sometimes true [2], often true [3], and true nearly all of the time [4]. Higher scores indicate higher resilience.

The scale demonstrated good psychometric properties and factor analysis yielded five factors: Personal competence, high standards, and tenacity; Trust in one’s instincts, tolerance of negative affect, and strengthening effects of stress; Positive acceptance of change and secure relationships; Control; Spiritual influences. The scale demonstrates that resilience is modifiable and can improve with treatment, with greater improvement corresponding to higher levels of global improvement.

- Childhood Trauma Questionnaire (CTQ) [35]

It is a 28-item self-report retrospective inventory intending to measure childhood or adolescent abuse and neglect. The CTQ contains five subscales, three assessing abuse (Emotional, Physical, and Sexual) and two assessing neglect (Emotional and Physical). A 5-point frequency of occurrence scale is used: [1] never true, [2] rarely true, [3] sometimes true, [4] often true, and [5] very often true. Each subscale score ranges from 5 (no history of abuse or neglect) to 25 (very extreme history of abuse and neglect).

- Post-traumatic Stress Disorder Checklist for DSM-V (PCL-5) [36]

It is a 20-item self-report measure that assesses the presence and severity of Post-traumatic Stress Disorder (PTSD) symptoms. A total symptom severity score ranges from 0-80. A 5-10 point change represents reliable change (i.e., not due to chance), a 10-20 point change represents a clinically significant change.

- Beck Hopelessness Scale (BHS) [37]

It is a 20-item self-report inventory that was designed to measure three major aspects of hopelessness.. The internal reliability coefficients are reasonably high (Pearson r= .82 to .93 in seven norm groups), but the BHS test-retest reliability coefficients are modest (.69 after one week and .66 after six weeks).

- Beck’s Suicide Intent Scale (SIS) [38]

It is a brief 21-item scale that assesses the person’s current intensity of attitudes, plans, and behaviors to commit suicide. The SSI examines the duration and frequency of ideation, the sense of control over an attempt, the number of deterrents, and the amount of planning involved in a contemplated attempt. Total scores range from 0 to 38. A 0-10 score suggests a low suicide risk; a 10-20 score a medium suicide risk; scores above 20 represent a high suicide risk.

- Brief Aggression Questionnaire (BAQ) [39]

It is a 12-item self-report measure of aggression with four subscales: Physical Aggression, Verbal Aggression, Anger, and Hostility.

- Deliberate Self-Harm Inventory (DSHI) [40]

It is a 17-item, behaviorally based, self-report questionnaire developed to assess deliberate self-harm. A continuous variable was created to measure the frequency of reported self-harm behavior.

### Statistical analysis

A descriptive analysis of the sample was carried out. Patients’ characteristics were summarized using absolute and relative frequencies for categorical variables and mean and standard deviation (SD) for continuous ones. To simplify the interpretation of data, the numeric results of the questionnaire were categorized using the cut-off suggested by the literature or clinical experience. In between-group comparisons were carried out for male and female subjects for psychiatric disorders using independent sample t-test (parametric test).

Pearson correlation matrices were constructed to investigate the association among variables. Pairwise t-tests were conducted and p values<0.05 were considered statistically significant.

The influence of socio-demographic factors and psychological features on post-traumatic stress disorder (as assessed with the PCL-5), hopelessness (as assessed with the BHS) and resilience (as assessed with the CD-RISC) was investigated via univariate and multivariate general linear models.

In the first step, separate ANOVA models were performed for each outcome (PCL score, BHS score, CD-RISC score) and p-values of F-tests were reported. Then, the testing of coefficients across equations was performed with multivariate analysis using the Wilks' lambda and the corresponding p-value s were reported. The independent variables were considered in a univariate way; a p-value <0.05 and p-value <0.001 were the cutoffs for statistical significance and for strong statistical significance.

The mediating and moderating analysis was conducted testing if CD-RISK, BHS and PCL total scores CD-RISK, BHS and PCL moderate or mediate the association between childhood trauma experiences (CTQ-28) and the clinical features assessed (SDS, SAS, BAQ, SIS). The mediation role was assessed by conducting three separate linear regressions for each combination of dependent, independent and possible mediator variables exposed before. Particularly, the three models were: i) a linear regression with the resilience factors as dependent variable and childhood trauma as the independent variable (term a), ii) a multiple regression using a saturated model in which the mediator (term b) in question and the childhood trauma (term c) were entered as independent variables with the appropriate measure of clinical features entered as the dependent variable, iii) a linear regression with clinical features as dependent variable and childhood trauma as the independent variable (term c). The data from the statistical analyses mentioned above were then used to calculate the z-value from Sobel’s test and the corresponding p-value. Evidence for mediation is said to be likely if the term a, c, b and Sobel’s test are significant.

The moderating role was estimated by performing a hierarchical regression in which the independent variable, CTQ, was entered in the first block, the possible moderator was entered in the second block, and lastly, the interaction variable (independent variable multiplied by the moderator variable) was entered in the third block. There is evidence of moderation if the interaction p-value is significant (p value < 0.05).

The SAS statistical software (version 9.4) was used for all analyses [41].

## Results

### Socio-demographic features

One hundred and nineteen migrants were recruited between May 2017 and April 2018 at NIHMP. The sample included 102 males (85.71%) and 17 females (14.29%); their mean age was 29.4 ± 10.52 (range 18-79), and the mean years spent in Italy at the time of enrolment was 3.96 ± 5.66 (range 0.08-30 years). Seventy-nine migrants (80.67%) were from Africa, mostly Western Africa (N= 52; 66.39%); 6.72% (N=8) were from Eastern Europe (predominantly from Romania, Poland and Moldova) and South Asia (predominantly from India, Pakistan, Bangladesh). The remaining 5.89% (N=7) were from other countries.

Educational level was the following: 12 migrants (12.61%) obtained graduation, 33 (27.73%) completed high school, 30 (25.21%) secondary school, 22 (18.49%) primary school, and only 15 (12.61%) were illiterate. As regards employment situation, two (1.68%) were students, 19 (15.97%) were employed; most were unemployed (N = 94, 78.99%), one was retired. Marital status was single for 86 (72.27%) migrants, 20 (16.81%) were married, 2 (1.68%) legally separated and 6 (5.04%) divorced, 4 (3.36%) were widows. Socio-economic condition was low for 89 (74.79%) migrants, medium for 17 (14.29%), high only for one (0.84%). This information was missing for 12 migrants. Fifty-eight (48.74%) received low social support, 42 (35.39%) high support, and for 19 (N= 19,97%) migrants there was no data about this issue. Religion was Muslim for 56 (47.06%), Christian for 48 (40.34%), other religion for 11 (9.24%). In other religions, 6 (N= 5.04%) Atheists, 1 (N= 0.84%) Adventist, 1 (N= 0.84%) Buddhist, 2 (N= 1.68%), Hindu, 1 (N= 0.84%) Jehovah’s Witness were included.

### Gender differences

Twenty-eight (37.8%) males suffered from adjustment disorder and trauma- and stressor-related disorders (vs N=3 females, 25%), 10 (13.51%) males from anxiety disorders, insomnia, somatic symptoms and related disorders or substance-related and addictive disorders (vs N=3, 25% females), 25 (33.78%) males from bipolar and related disorders, depressive disorder, schizophrenia spectrum and other psychotic disorders (vs N=4, 33.3% females). Eleven (14.86%) males didn’t receive a psychiatric diagnosis (vs N=2, 16.67% females). For 5 migrants, psychiatric diagnosis data was missing. The most common psychiatric disorder among the two samples was adjustment disorder and trauma- and stressor-related disorders . Globally, there was not observed a statistical significance difference (p<0.05), due to the inhomogeneities of the sample (74 males and 12 females).

### Clinical features

**Fig. 1:**
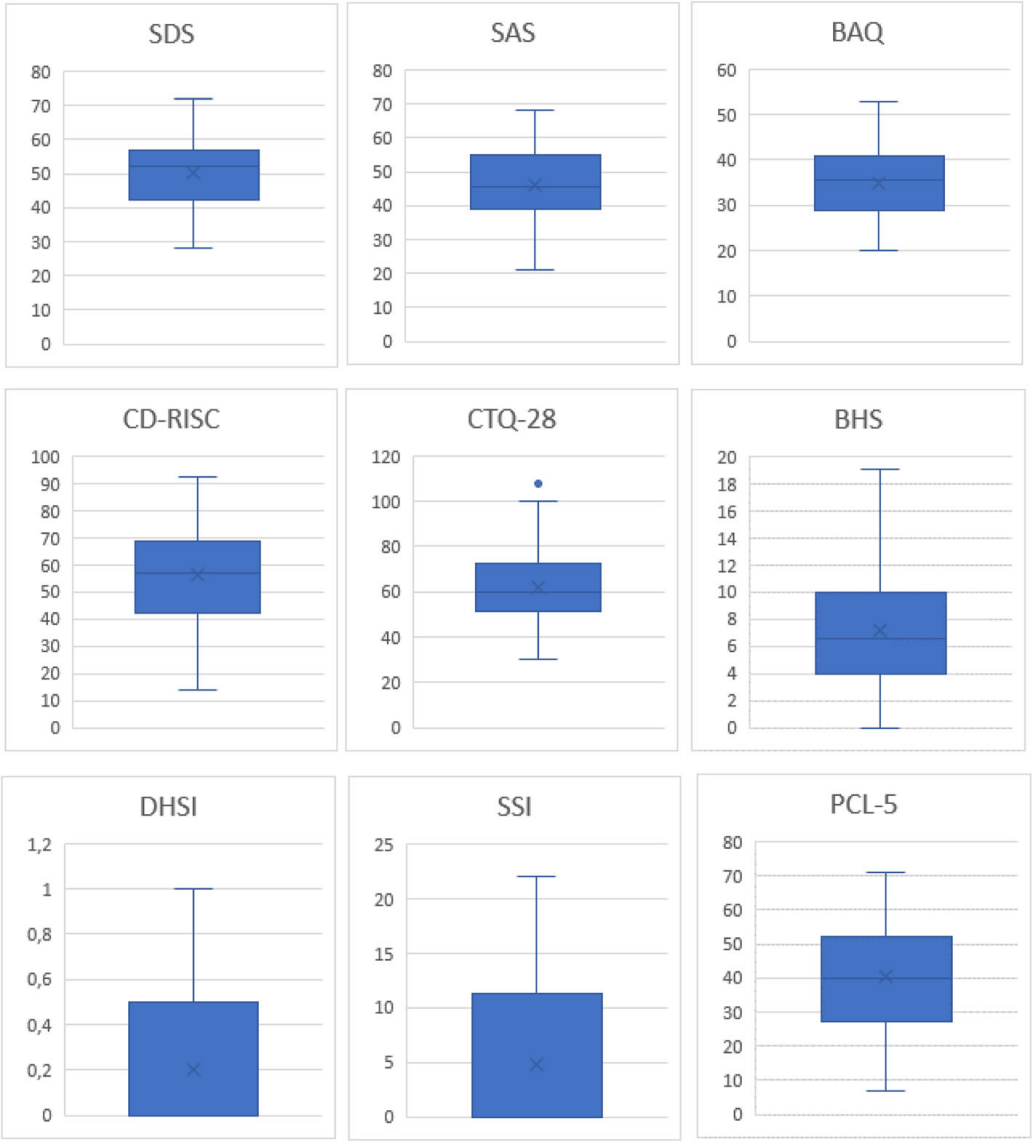
Box blot different questionnaires. Zung Self rating Depression Scale (SDS) = min 28- max 77; Zung Self rating Anxiety Scale (SAS) = min 21- max 69; Brief Aggression Questionnaire (BAQ) = min 17- max 53; Childhood Trauma Questionnaire (CTQ-28) total score = min 28-max 108; CTQ-28 Emotional maltreatment = min 4- max 25; CTQ-28 Physical maltreatment = min 5- max 25; CTQ-28 Sexual Abuse = min 4 – max 25; CTQ-28 Emotional neglect = min 4 – max 25; CTQ-28 Physical neglect = min 5 –max 21; PTSD Checklist for DSM-V (PCL-5) = min 7- max 71; Scale for Suicide Ideation (SSI) = min 0 – max 22; Beck Hopelessness Scale (BHS) = min 1 – max 20; Deliberate Self Harm Inventory (DSHI) = min 0 – max 1

71.43% (N = 85) of the subjects reported a history of traumatic experiences during adulthood, especially imprisonment and physical violence (respectively 17.17%, N = 16 and 13.13%, N = 11). 80.67% (N = 96) of migrants reported a history of stressful life-events, 14.29% (N = 17) presented agitation at the moment of the survey; 70.59% (N = 84) declared to suffer from insomnia and 42.86% (N = 51) from social isolation. The consumption of alcohol and drugs was reported by 8.40% (N = 10) of migrants’ sample. Thirty patients (25.21%) had a current medical diagnosis. A score above the PCL-5 33-points cut-off was found in 53.39% (N = 63) of migrants; overall, the mean score was 39.45. Depression symptoms as assessed with the SDS were below the cutoff in 42.37% (N = 50) of migrants (mean score of the whole sample: 50.31). SAS scores in 38.98% (N = 46) were indicative of low anxiety. Regarding the CTQ, physical maltreatment and neglect were reported by 56.78% (N = 67) and 69.49% (N = 82) of migrants, respectively. SIS scores suggested a low, medium and high suicide risk in 74.77%(N = 83), 12.61% (N = 14)and 7.21% (N = 8) of the sample, respectively. See Table 1 and Fig. 1 for further details about the scores of the questionnaires.

**Table 1:** Summary of the results of the questionnaire (Zung Self rating Depression Scale (SDS); Self rating Anxiety Scale (SAS); Childhood Trauma Questionnaire (CTQ-28) PTSD Checklist for DSM-V (PCL-5); Beck Hopelessness Scale (BHS), Beck’s Suicide Intent Scale (SIS) using absolute and relative frequencies

	N	%
**SDS**		
*Missing*	4	3.39
*No depression*	50	42.37
*Low depression*	45	38.14
*Mild depression*	17	14.41
*High depression*	2	1.69
**SAS**		
*Missing*	3	2.54
*No anxiety*	46	38.98
*Low anxiety*	58	49.15
*Mild anxiety*	11	9.32
**CTQ-28 emotional maltreatment**		
*Missing*	6	5.08
*< 13*	68	57.63
*> =13*	44	37.29
**CTQ-28 physical maltreatment**		
*Missing*	6	5.08
*< 10*	45	38.14
*> =10*	67	56.78
**CTQ-28 sexual abuse**		
*Missing*	6	5.08
*< 8*	60	50.85
*> =8*	52	44.07
**CTQ-28 emotional neglect**		
*Missing*	6	5.08
*< 15*	64	54.24
*> =15*	48	40.68
**CTQ-28 physical neglect**		
*Missing*	6	5.08
*< 10*	30	25.42
*> =10*	82	69.49
**PCL-50**		
*Missing*	26	22.03
*< 33*	29	24.58
*> =33*	63	53.39
**BHS**		
*Missing*	6	5.08
*0–3*	17	14.41
*4–8*	54	45.76
*9–14*	31	26.27
*15–20*	10	8.47
*> =10*	82	69.49
**SIS**		
*Missing*	6	5.40
*0–10*	83	74.77
*11–20*	14	12.61
*20–38*	8	7.21

More specifically, concerning self-harm behavior and suicide attempt, 10.92% (N = 13) of migrants reported a lifetime history of at least one suicide attempt. 7.56% (N = 9) of migrants had a family member who died for suicide; 7.56% (N = 9) reported a family history of suicide attempt, and 7.87% (N = 7) a family history of psychiatric disorders. Pearson’s Correlation analysis highlighted several positive statistically significant, and strongly significant correlations among the study variables (See Table 2 for details). A negative correlation (p < 0.05) was found between CD-RISC and SDS and between CD-RISC and SSI. Table 3 shows the results of a separate ANOVA and multivariate models that analyze the influence of socio-demographic factors and psychological features on post-traumatic stress disorder (as assessed with the PCL-5), hopelessness (as assessed with the BHS) and resilience (as assessed with the CD-RISC).The number of subjects, mean and standard deviation, beta, and 95% confidence interval, and discussed in the section below Age did not influence the scores obtained at PCL-5, BHS, CD-RISK.

**Table 2:** **Pairwise pearson’s** Correlation between SDS, SAS, BAQ, CD-RISC, CTQ-28, PCL-5,SSI, BHS scores, and p-values. It are reported also the number of subjects in each pairwise evaluation

	SDS	SAS	BAQ	CD-RISC	CTQ_28	PCL_5	SSI	BHS
SDS n=	1	0.78*	0.40*	−0.24*	0.27	0.53*	0.30*	0.49*
	114	114	112	113	112	92	112	111
SAS n=		1	0.55*	−0.15	0.30*	0.55*	0.31	0.47*
		115	112	113	112	92	112	112
BAQ n=			1	−0.10	0.30*	0.45*	0.36*	0.26
			113	112	111	92	111	110
CD-RISC n=				1	−0.16	0.10	−0.31*	−0.46*
				113	112	92	112	111
CTQ_28 n=					1	0.25*	0.21*	0.21*
					112	92	111	110
PCL-5 n=						1	0.17	0.21*
						92	91	90
SSI n=							1	0.34
							112	110
BHS n=								1
								112

P* < 0.05

**Table 3:** Number, mean, standard deviation and ANOVA’s p-values of PCL-5, BHS and CD-RISC scores specified for different sociodemographic and clinical variables, and SDS, SAS, CTQ scores. We reported also, in the last column, the p-values of Multivariate analysis

	PCL (n = 93)				BHS (n = 113)				***CD-RISK (***n = 114***)***				***MANOVA (n = 90)***
	***N***	***Mean***	***STD***	***p-value***	***N***	***Mean***	***STD***	***p-value***	***N***	***Mean***	***STD***	***p-value***	***p-value***
**Sex**													
*Female*	13	39.15	18.2	0.90698	16	7.06	3.91	0.77880	16	57.38	19.05	0.94257	0.98107
*Male*	80	39.69	14.69		97	7.74	4.68		98	54.65	19.23		
**Age**													
*< 25 yrs*	48	37.90	13.67	0.53490	51	6.82	3.62	0.30857	52	55.96	20.00	0.39312	0.53490
*> 25 yrs*	45	41.44	16.48		62	8.32	5.16		62	54.26	18.53		
**Geographic Area**													
*Europe*	6	36.17	15.72	0.78925	10	5.4	3.86	0.14143	10	61.3	19.88	0.54794	0.19945
*Asia*	6	39	15.27		12	11.83	4.75		12	51.33	20.24		
*Africa*	72	40.25	14.05		82	7.32	4.27		83	54.46	19.42		
**Education level**													
*Low*	11	36.09	11.41	0.29901	13	9.62	4.35	0.71730	13	54.23	19.75	0.61421	0.33339
*Medium*	42	42.55	16.06		51	7.18	3.65		50	53.62	19.73		
*High*	36	37.94	15.02		44	7.91	5.42		45	56.11	19.59		
**Employment situation**													
*Unemployed*	74	40.57	14.96	0.35970	94	7.83	4.59	0.84788	94	54.67	20.12	0.89253	0.84192
*Worker*	18	36.5	15.79		17	7.35	4.34		18	55.39	14.08		
**Marital status**													
*Single*	67	39.31	15.12	0.36713	83	7.57	4.62	0.65205	84	54.36	18.35	0.41177	0.26782
*Married*	17	43.41	12.92		19	7.37	4.25		19	56.68	19.99		
*Divorced /Widow*	9	34.67	18.72		11	8.73	5		11	57.36	24.79		
**Religion**													
*Muslim*	44	40.34	15.09	0.44680	54	8.96	4.61	0.00049**	54	50.81	18.94	0.02555*	0.00526*
*Christian*	40	38.35	14.72		45	5.8	3.89		47	59.17	18.58		
*Other*	7	46.14	17.93		11	8.64	4.86		11	57	21.3		
**Psychiatric disorder**													
*No*	13	31.69	13.82	0.00703**	14	4.43	2.14	0.04999*	14	68.36	14.93	0.07017	0.00764*
*Traumatic*	33	41.12	14.41		37	8.38	4.87		39	50.08	17.19		
*Neurotic*	13	30.54	19.85		19	6.89	3.83		18	53.83	17.71		
*Psychotic*	29	44.86	11.8		37	8.27	4.95		37	54.86	20.83		
**Social support**													
*Low*	37	39.3	17.11	0.97496	40	6.83	4.16	0.97661	42	60.02	16.63	0.08350	0.29070
*High*	45	39.18	13.13		57	8.05	4.63		57	51.95	18.98		
**Socio-economic condition**													
*Low*	70	40.09	15.13	0.49704	88	7.85	4.6	0.60908	87	53.86	19.29	0.29750	0.31511
*Medium-High*	14	37.57	16.11		17	6.65	4.6		18	62.06	18.76		
**Traumas in adult life**													
*No*	17	37.41	18.33	0.56293	20	6.35	4.38	0.83535	20	60	16.64	0.60848	0.87057
*Yes*	68	39.82	14.32		80	7.9	4.63		82	53.2	19.24		
**Negative stressful events**													
*No*	11	35.55	14.26	0.36411	13	5.77	4.49	0.39757	13	61.85	18.94	0.73768	0.72667
*Yes*	75	39.91	15.14		93	7.92	4.62		94	54.04	19.37		
	**PCL (n = 93)**				**BHS (n = 113)**				**CD-RISK (n = 114)**				**Multivariate p-values (n = 90)**
	***N***	***Mean***	***STD***	p-value	***Mean***	***STD***	***N***	***p-value***	***N***	***Mean***	***STD***	***p-value***	
**Agitation**													
*No*	63	39.43	15.27	0.22521	79	7.15	4.44	0.79012	81	57.2	18.8	0.60715	0.58751
*Yes*	16	44.56	12.66		17	7.12	4.23		17	54.35	18.19		
**Insomnia**													
*No*	23	31.26	14.29	0.00116**	24	6.13	3.86	0.29766	25	56.72	20.23	0.75819	0.01391*
*Yes*	65	42.6	14.68		83	7.81	4.57		84	54.95	18.76		
**Social isolation**													
*No*	42	37.31	16.29	0.13833	47	7.38	4.44	0.20579	49	55.51	18.7	0.70292	0.14515
*Yes*	39	42.31	13.47		51	7.57	4.67		51	54.29	20.34		
**Current physical diagnosis**													
*No*	65	38.98	15.78	0.43230	80	7.03	4.43	0.01934*	81	57.49	18.28	0.70292	0.11402
*Yes*	24	42.21	13.88		28	9	4.57		29	48.9	19.82		
**Suicide attempt**													
*No*	75	39.11	14.62	0.15806	90	7.16	4.46	0.16643	91	56.11	19.83	0.64612	0.34585
*Yes*	8	47.13	18.28		13	10.62	4.74		13	47.31	15.5		
**Zung Self rating Depression Scale (SDS)**													
*No depression*	41	32.8	14.04	0.00014**	51	5.57	3.66	0.00002**	51	60.53	19.37	0.01603*	0.00000**
*Mild depression*	40	43.48	14.45		43	8.09	4.05		45	52.02	18.19		
*Moderate-high depression*	12	50	10.44		18	12.33	4.56		18	47	17.18		
**Zung Self rating Anxiety Scale (SAS)**													
*No anxiety*	37	29.81	12.98	0.00000**	47	5.68	3.37	0.00057**	46	55.41	19.66	0.53741	0.00000**
*Mild anxiety*	47	44.79	12.44		55	8.36	4.72		57	55.84	18.97		
*Moderate anxiety*	9	52.89	13.44		11	12.45	3.86		11	49.27	18.68		
**CTQ-28 Emotional maltreatment**													
*Low*	56	36.95	14.84	0.02769*	67	7.18	4.34	0.92425	68	54.19	20.69	0.40690	0.15812
*Medium-High*	37	43.65	14.83		44	8.43	4.86		45	56	16.83		
**CTQ-28 Physical maltreatment**													
*Low*	39	35.05	14.89	0.00942**	44	7.43	4.84	0.88137	45	55.82	22.1	0.84046	0.06007
*Medium-High*	54	42.91	14.54		67	7.84	4.42		68	54.31	17.14		
**CTQ-28 Sexual abuse**													
*Low*	49	37.53	17.36	0.16027	60	7.38	4.6	0.07472	60	55.77	19.88	0.43309	0.22764
*Medium-High*	44	41.93	11.93		51	8.02	4.57		53	53.94	18.51		
**CTQ-28 Emotional neglect**													
*Low*	55	41.24	16.46	0.21480	62	6.61	3.94	0.01912*	64	62.77	17.54	0.00000**	0.00000**
*Medium-High*	38	37.26	12.78		49	9.02	4.99		49	44.65	16.29		
**CTQ-28 Physical neglect**													
*Low*	23	33	17.5	0.01015*	29	7.28	5.3	0.14296	30	61.87	20.32	0.17732	0.02475*
*Medium-High*	70	41.79	13.7		82	7.82	4.32		83	52.4	18.24		

**Fig. 2:**
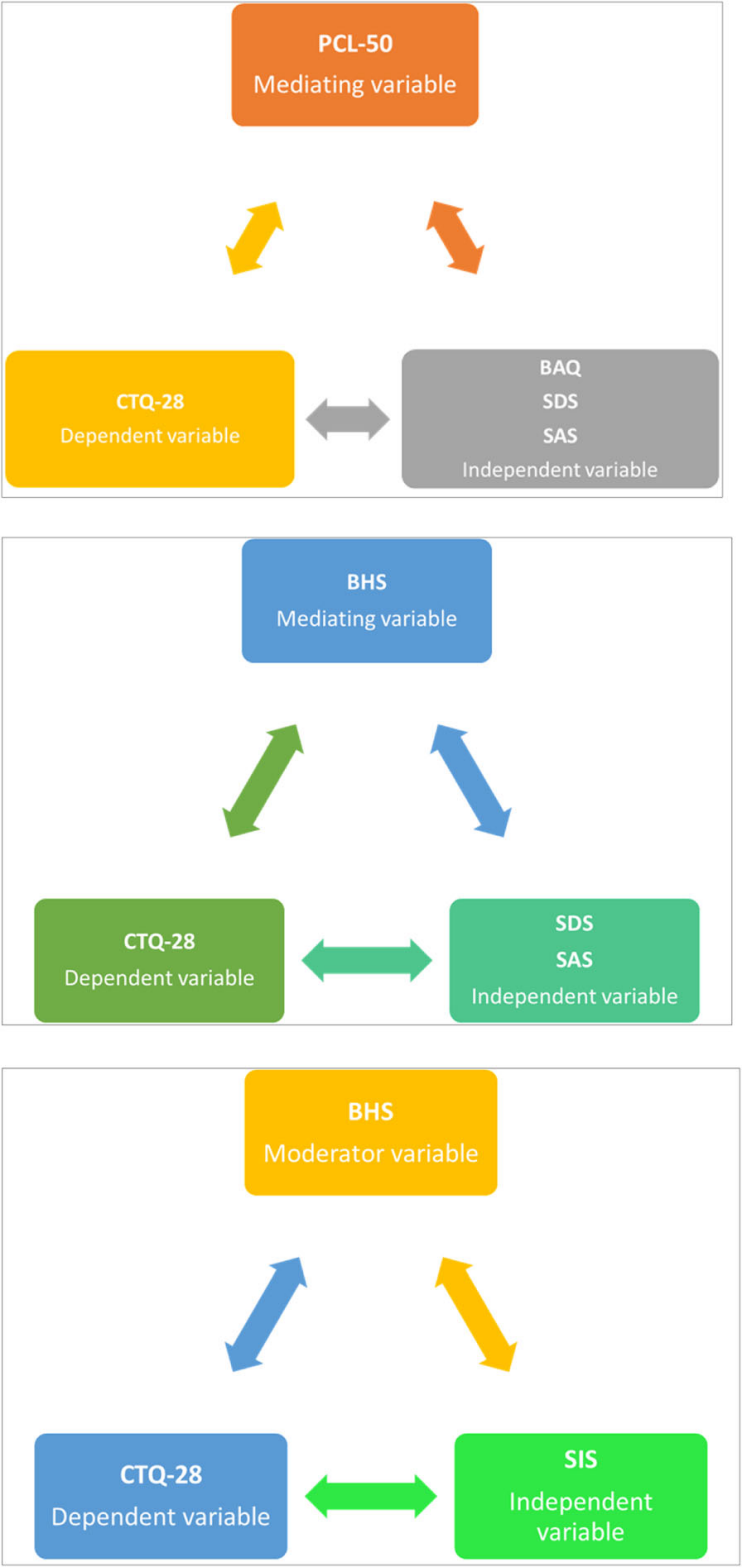
Mediating and moderating role of CD-RISK, BHS and PCL in the relationship between CTQ-28 and clinical features (SDS, SAS, BAQ, SIS)

Finally, Table 4 and Fig. 2 presents data about the mediating and moderating role of resilience factors (CD-RISK), hopelessness (BHS) and PTSD symptoms (PCL-5) in the relationship between Childhood Trauma and clinical features (SDS, SAS, BAQ, SIS). PCL-50 resulted to be a mediator factor in the relationship between CTQ-28 and BAQ (p-value = 0.03538), SAS (p-value = 0.02505), SDS (0.02613). BHS resulted to a mediator factor in the relationship between CTQ-28 and SDS (p value = 0.04842) and SAS (p value = 0.05046), while resulted a moderator factor in the relationship between CTQ-28 and BHS (p-value = 0.037).

**Table 4:** Assessment of mediating and moderating role of CD-RISK, BHS and PCL in the relationship between Childhood Trauma (CTQ-28) and clinical features (SDS, SAS, BAQ, SIS). Evidence of moderation if the interaction p-value is significant (p < 0.05)

X (totale score)	Y (total score)	M (total score)	p-Value Sobel Test	p-Value Interaction Model
CTQ 28	BAQ	BHS	0.12949	0.267
CTQ 28	BAQ	CD RISC	0.56490	0.757
CTQ 28	BAQ	PCL 5	0.03538*	0.736
CTQ 28	SAS	BHS	0.05046*	0.961
CTQ 28	SAS	CD RISC	0.38120	0.877
CTQ 28	SAS	PCL 5	0.02505*	0.759
CTQ 28	SDS	BHS	0.04842*	0.566
CTQ 28	SDS	CD RISC	0.18681	0.894
CTQ 28	SDS	PCL 5	0.02613*	0.076
CTQ 28	SIS	BHS	0.06834	0.037*
CTQ 28	SIS	CD RISC	0.13911	0.251
CTQ 28	SIS	PCL 5	0.29913	0.592

## Discussion

The adverse impact of migration on mental health is widely acknowledged by the existing literature [42, 43], which points to a multifaceted causation related to the exposure to traumatic events, daily stressors, and impoverishment [44]. A systematic review of resilience and mental health outcomes [3] showed that migration-related stress, together with post-migration adverse environments, trauma exposure and pre-existing vulnerabilities, may predict the development of psychopathology such as depression, anxiety and PTSD [42, 43, 45, 13]. Our current research sheds new light not only on trauma, but also on hopelessness and resilience in a migrant sample, independently from a forced or traumatic process of migration.

In this study, scores suggesting PTSD were found in 53.39% of migrants; mild to high depressive symptoms according to SDS scores and mild anxiety symptoms according to SAS scores emerged in 16.1 and 9.32% of the sample, respectively.

Consistent with previous researches which highlighted that migrants scored high on anxiety, depression, childhood trauma, aggression, and impulsivity measures, and also reported frequent suicide attempts and self-mutilation behaviors [46], our study found correlations between suicidal intent and anxiety, depression and aggressiveness (as pointed out by Pearson’s correlation among SSI, SAS, SDS and BAQ).Regarding resilience, which we assessed with the CD-RISC questionnaire, we found several interesting results, consistent with clinical observations. First, lower resilience was correlated with higher levels of hopelessness in migrants. Furthermore, resilience was inversely correlated to anxiety and suicide intent as assessed with the SAS and SIS, respectively. The mediation role of resilience on mental health outcomes is not entirely clear. Indeed, resilience is considered both a protective factor and not, related to positive or negative psychological outcomes [47]. While the impact of factors such as employment status, debt or food security on mental health is clearly established [3, 42, 48, 49], more research is warranted to analyze the mental health-resilience nexus.

Our study found that more resilient migrants had higher levels of psychological well-being. Surprisingly, resilience was higher among migrants without social support. This trend is in contrast with the international literature that indicates that social support is essential for maintaining physical and psychological health [50, 51]. An explanation of this observation is the possibility that resilience - the ability to manage and recover from life’s challenges – may be a key differentiator in situational management coping. Resilient migrants may ask for help when they are in need and may use the available resources, limiting an exaggerated use or overuse of social support service. Taking a number of other factors into account in psychological resilience nonetheless it is likely that social support is not universally helpful; moreover, the effectiveness and the extent of social support depend on individual needs, which may change over time [52].

Regarding trauma, we measured childhood or adolescent abuse and neglect and the presence and severity of PTSD symptoms, respectively with the CTQ and PCL-5 questionnaires. In accordance with other authors [26, 53, 54], we found that PTSD was frequent among migrants, while depressive symptoms were less common. Nonetheless, the survey design did not include a control native population, did not focus on either second or third generation migrants, or on refugees/asylum seekers. However, this could be a strength of the study because while the history of traumatic events is very common in selected populations of immigrants like refugees and asylum seekers, previous findings on immigrants accessing primary care are conflicting. Hence our results might add to the existing knowledge in this field of research.

Migration can be connoted by multiple traumatic events (such as experiences of loss, interpersonal trauma, and life stress), exposing the migrant population to a higher risk of PTSD [53, 55–57]. and depression [57, 58]. The findings of this study support the idea [59] that PTSD is often associated with anxiety and depression. It often happens that premigration experiences, individual development and the nature of trauma or post-trauma reactions are not adequately explored; furthermore, migrants’ experiences are heterogeneous, and responses to the process of migration may be varying.

As described in the introduction, the literature about childhood trauma in migrants is still scant. In our study, less than half of migrants reported a history of childhood maltreatment (mean CTQ-28 score was 60.9, slightly below the materiality 61-points threshold). However, childhood physical maltreatment was reported by 56.78% of the sample, and childhood physical neglect by 69.49%. Actually, a recent review and meta-analysis [60] did not provide evidence for higher risk of child maltreatment in immigrants or refugees, while highlighting that the recently settled immigrants and refugees may experience specific stressors and challenges, which may result in high risk of maltreatment.

Moreover, in our sample childhood trauma was associated with hopelessness and PTSD; particularly, physical and emotional maltreatment and physical neglect were associated with PTSD, while emotional neglect was positively correlated with hopelessness and low resilience. Despite the general lack of research about adverse childhood experiences in migrants limits the possibility to compare the current results with those by other authors, we have found that the diagnosis of mood, anxiety, personality and / or substance use disorders, as well as the likelihood of violent and non-violent antisocial behavior, are more related to having suffered a limited adverse experiences, abuse emotional and physical, family violence, as well as global adversities [61–63]. In addition, it is widely acknowledged that childhood trauma and aggression are distal risk factors predisposing to the development of suicidal behavior [64–66]. Previous researches [67, 68] suggested that the severity of chronic anxiety, aggression (particularly hostility), and impulsivity were mediators of the association between childhood trauma and dissociation.

Finally, mediator and moderator analyses have shown that PTSD symptoms play the role of mediators for the relation between the childhood traumatic experiences and aggressiveness, anxious and depressive symptomatology, while hopelessness is a mediator between the childhood traumatic experiences and the development of depression or anxiety in adulthood. Hopelessness seems to influence the strength of the relation between childhood traumatic experiences and the individual’s current intensity of suicidal attitudes, plans, and behaviors.

Analyzing possible correlations of PTSD, hopelessness, and resilience with other socio-demographic and clinical characteristics, it resulted that African, Asian and Muslims migrants reported higher levels of hopelessness, while Christians were more resilient than other religious groups. Age does not seem to affect the scores obtained at PCL-5, BHS, CD-RISK, probably because in our sample there are a majority of young people or adults.

Patients with psychotic disorders reported highest scores on the PCL-5 (44.86), followed by patients with traumatic disorders (41.12) and neurotic disorders (30.54). Generally, patients affected by psychosis showed higher scores on the hopelessness and resilience scales. Hopelessness is higher among patients with traumatic disorders, followed by psychotic disorders.

PCL-5 scores showed a strong correlation with anxiety, depression, insomnia and childhood physical maltreatment and a correlation with childhood emotional maltreatment and physical neglect.

These results have no unique explanation. In fact, the strengthening of resilience is not based on the avoidance of stressful factors but on the ability to count on self-confidence and social support when exposed to traumatic or intense stressful events. Resilience seems to be conditioned by events concerning different phases of life (later childhood, adolescence, adult life), which, when combined, might create a network of indirect protective links to adverse events.

Moreover, our results seem to support the importance of the development of depression-focused services or programs for the migrant population, aimed at providing preventive strategies reducing stress and depression-, especially for people with high levels of hopelessness.

### Strengths and limitations

The strengths of the study include large participation at baseline, which grants low bias. Moreover, the current study is one of the few analyzing resilience and trauma in a population of migrants using a specific tool, composed by different questionnaires, and taking into consideration numerous factors, including demographic, economic and social variables. Further developments and future perspectives of this research project could be addressing key gaps in the field of resilience research by means of a longitudinal evaluation study in the adult population of migrants, including a control group from the native population, acceding to NIHMP.

Mediator and moderator analyses were conducted to show the possible role of resilience, hopelessness and PTSD as mediators or moderators between traumatic experiences in childhood and the development of psychiatric symptomatology in adulthood.

Clearly, migrant studies may be affected by cultural biases. Regarding the sampling method and sample size, they do not allow to assume that the sample recruited was representative of the general population. Other limitations of the study are the inclusion criterion of adequate knowledge of either Italian or English language, the lack of data concerning the subjects who refused to participate, and the absence of a control population of native individuals. Last, several data were missing but were handled using a combination of complete case analysis and last observation carried forward methods.

A further limitation is the lack of a thorough assessment of the migration history and of the traumas that could have occurred during the migration phase, with important consequences on migrants’ mental health.

## Conclusions

Our findings indicate that migrants with anxiety disorders or depressive disorders suffered from more psychiatric comorbidities than those with other diagnoses; furthermore, they were more hopeless and less resilient, and more likely to have post-traumatic stress disorders.

A longitudinal study on resilience and trauma among migrant populations seems to be really useful in understanding the temporal interactions of these two factors and their contribution in developing mental health disorders, or vice versa their protective role in preventing psychopathology.

Overall, the baseline data of this longitudinal research project on resilience and trauma, seem to suggest the importance of enhancing mental health at the primary care level, and developing evidence-based interventions focused on individuals and communities. For this purpose, it appears to be of fundamental importance to favor the development of researches that analyze resilience for the development of targeted interventions.

Future aims of this ongoing research project include the recruitment and follow-up of a larger sample, and the assessment of migratory and cultural factors, encouraging identification and treatment of child maltreatment and its consequences in adult life. Moreover, further studies are clearly needed to determine whether the migration process could be related to the development of mental disorders in migrant populations, during resettlement, taking into consideration pre-migration, migration and post-migration traumas, and stress conditions. Further researches on migrant resilience with a focus on strengths and abilities, external supports, incorporating strengths-based approaches are needed to reinforce resilience.

## Data Availability

The datasets used and/or analyzed during the current study are available from the corresponding author on reasonable request.

## Abbreviations

PTSD: Post-Traumatic Stress Disorder
NIHMP: The National Institute For Health, Migration And Poverty
SDS: Zung Self-Rating Depression Scale
SAS: Zung Self-Rating Anxiety Scale
CD-RISC: Connor-Davidson Resilience Scale
CTQ: Childhood Trauma Questionnaire
PCL-5: Posttraumatic Stress Disorder Checklist For DSM-V
BHS: Beck Hopelessness Scale
SIS: Beck’s Suicide Intent Scale
BAQ: Brief Aggression Questionnaire
DSHI: Deliberate Self-Harm Inventory
SD: Mean And Standard Deviation

## References

[1] PRIEBE S, GIACCO D, EL-NAGIB R. Public health aspects of mental health among migrants and refugees: A review of the evidence on mental health Care for Refugees, asylum seekers and irregular migrants in the WHO European region [internet]. Copenhagen: WHO Regional Office for Europe; 2016. (Health Evidence Network Synthesis Report, No. 47.) Available from: https://www.ncbi.nlm.nih.gov/books/NBK391045/. 27809423

[2] SIRIWARDHANA C, ABAS M, SIRIBADDANA S (2015). Dynamics of resilience in forced migration: a 1-year follow-up study of longitudinal associations with mental health in a conflict-affected, ethnic Muslim population. BMJ Open.

[3] SIRIWARDHANA C, ALI SS, ROBERTS B, STEWART R (2014). A systematic review of resilience and mental health outcomes of conflict-driven adult forced migrants. Confl Health.

[4] AHMED K, BHUGRA D (2007). Depression across ethnic minority cultures: diagnostic issues. World Cultural Psychiatry Research Review.

[5] LAZARUS RS, FOLKMAN S (1984). Stress, appraisal, and coping.

[6] KUO BC (2014). Coping, acculturation, and psychological adaptation among migrants: a theoretical and empirical review and synthesis of the literature. Health Psychol Behav Med.

[7] BUSTAMANTE LHU, CERQUEIRA RO, LECLERC E, Brietzke E, Lhu B, Ro C, Leclerc E, Brietzke E (2017). Stress, trauma, and posttraumatic stress disorder in migrants: a comprehensive review. Braz J Psychiatr.

[8] CASTRO FG, MURRAY KE, Reich JW, Zautra AJ, Hall JS (2010). Cultural adaptation and resilience: controversies, issues, and emerging models. Handbook of adult resilience.

[9] MARSIGLIA FF, KULIS S (2009). Diversity, oppression, and change: culturally grounded social work.

[10] MASTEN AS, MASTEN AS, ROLF J, CICCHETTI D, NUECHTERLEIN K, WEINTRAUB S (1990). Risk and protective factors in the development of psychopathology.

[11] RUTTER M. Family and school influences on cognitive development. J Child Psychol Psychiatry 1985; 26:683–704. 10.1111/j.1469-7610.1985.tb00584.x 3900115

[12] KAPLAN H.B. Toward an Understanding of Resilience. In: Glantz M.D., Johnson J.L. (eds) Resilience and Development. Longitudinal Research in the Social and Behavioral Sciences: An Interdisciplinary Series. Springer, Boston, MA; 2002.

[13] CLOSE C, KOUVONEN A, BOSQUI T, PATEL K, O'REILLY D, DONNELLY M (2016). The mental health and wellbeing of first generation migrants: a systematic-narrative review of reviews. Glob Health.

[14] MISSINNE S, BRACKE P (2012). Depressive symptoms among immigrants and ethnic minorities: a population based study in 23 European countries. Soc Psychiatry Psychiatr Epidemiol.

[15] İNCE BÜ, FASSAERT T (2014). DE wit MATTY. CUIJPERS P The relationship between acculturation strategies and depressive and anxiety disorders in Turkish migrants in the Netherlands BMC psychiatry.

[16] JUHASZ G, ESZLARI N, PAP D, GONDA X (2012). Cultural differences in the development and characteristics of depression. Neuropsychopharmacol Hung.

[17] ANTONIADES J, MAZZA D, BRIJNATH B (2014). Efficacy of depression treatments for immigrant patients: results from a systematic review. BMC Psychiatry.

[18] BHUGRA D et al, EPA guidance mental health care of migrants. Eur Psychiatry 2014;29(2):107–115. 10.1016/j.eurpsy.2014.01.003 24503244

[19] SIRIWARDHANA C, STEWART R (2013). Forced migration and mental health: prolonged internal displacement, return migration and resilience. Int Health.

[20] ABELA JR, PARKINSON C, STOLOW D, STARRS C (2009). A test of the integration of the hopelessness and response styles theories of depression in middle adolescence. J Clin Child Adolesc Psychol.

[21] BECKER-WEIDMAN EG, REINECKE MA, JACOBS RH, MARTINOVICH Z, SILVA SG, MARCH JS (2009). Predictors of hopelessness among clinically depressed youth. Behav Cogn Psychother.

[22] HAN XY, SHEK DT (2012). Socio-demographic and family correlates of hopelessness among adolescents in Shanghai, China. International Journal on Disability and Human Development.

[23] STEIN GL, GONZALEZ LM, HUQ N (2012). Cultural stressors and the hopelessness model of depressive symptoms in Latino adolescents. Journal of Youth and Adolescence.

[24] COURTNEY EA, JOHNSON JG, ALLOY LB (2008). Associations of childhood maltreatment with hopelessness and depression among adolescent primary care patients. Int J Cogn Ther.

[25] BISSON JI, COSGROVE S, LEWIS C, ROBERTS NP. Post-traumatic stress disorder. BMJ. 2015;351:h6161. Published 2015 Nov 26. doi:10.1136/bmj.h6161. 10.1136/bmj.h6161 PMC4663500 26611143

[26] LINDERT J, EHRENSTEIN OS, PRIEBE S, MIELCK A, BRÄHLER E. Depression and.

[27] anxiety in labor migrants and refugees--a systematic review and meta-analysis.Soc Sci Med. 2009 Jul;69(2):246–57. doi: 10.1016/j.socscimed.2009.04.032. 10.1016/j.socscimed.2009.04.032 19539414

[28] RASMUSSEN A, CRAGER M, BASER RE, CHU T, GANY F (2012). Onset of posttraumatic stress disorder and major depression among refugees and voluntary migrants to the United States. J Trauma Stress.

[29] VAN DER, VEGT EJ, TIEMAN W, VAN DER ENDE J, FERDINAND RF, VERHULST FC, TIEMEIER H (2009). Impact of early childhood adversities on adult psychiatric disorders: a study of international adoptees. Soc Psychiatry Psychiatr Epidemiol.

[30] HUTCHINSON M, DORSETT P (2012). What does the literature say about resilience in refugee people?. Implications for practice Journal of Social Inclusion.

[31] BABATUNDE-SOWOLE O, POWER T, JACKSON D, DAVIDSON PM, DIGIACOMO M (2016). Resilience of African migrants: an integrative review. Health Care Women Int.

[32] CONNOR KM, DAVIDSON JR (2003). Development of a new resilience scale: the Connor-Davidson resilience scale (CD-RISC). Depress Anxiety.

[33] ZUNG WW (1965). A self-rating depression scale. Arch Gen Psychiatry.

[34] ZUNG WW (1971). A rating instrument for anxiety disorders. Psychosomatics..

[35] VAN DER, KOLK BA, PERRY JC, HERMAN JL (1991). Childhood origins of self-destructive behavior. Am J Psychiatry.

[36] BERNSTEIN DP et al. Initial reliability and validity of a new retrospective measure of child abuse and neglect. Am J Psychiatry 1994, 151(8):1132–1136. 10.1176/ajp.151.8.1132 8037246

[37] BLEVINS CA, WEATHERS FW, DAVIS MT, WITTE TK, DOMINO JL (2015). The posttraumatic stress disorder checklist for DSM-5 (PCL-5): development and initial psychometric evaluation. J Trauma Stress.

[38] BECK AT, WEISSMAN A, LESTER D, TREXLER L (1974). The measurement of pessimism: the hopelessness scale. J Consult Clin Psychol.

[39] BECK AT, KOVACS M, WEISSMAN A (1979). Assessment of suicidal intention: the scale for suicide ideation. J Consult Clin Psychol.

[40] WEBSTER GD et al. The brief aggression questionnaire: psychometric and behavioral evidence for an efficient measure of trait aggression. Aggress Behav 2014, 40(2):120–139. 10.1002/ab.21507 24115185

[41] SANSONE RA, SANSONE LA (2010). Measuring self-harm behavior with the self-harm inventory. Psychiatry (Edgmont).

[42] SAS INSTITUTE INC. 2011. Base SAS® 9.3 Procedures Guide. Cary, NC: SAS Institute Inc.

[43] PORTER M, HASLAM N (2005). Predisplacement and postdisplacement factors associated with mental health of refugees and internally displaced persons: a meta-analaysis. JAMA..

[44] BHUGRA D. Migration and mental health. Acta Psychiatrica Scandinavia 2004, 109:234–258. 10.1046/j.0001-690x.2003.00246.x 15008797

[45] MILLER K, RASMUSSEN A (2010). War exposure, daily stressors, and mental health in conflict and post-conflict settings: bridging the divide between trauma-focused and psychosocial frameworks. Soc Sci Med.

[46] THOMAS S. Displacement and health. Br Med Bull 2004, 69:115–127. 10.1093/bmb/ldh009 15226201

[47] EVREN C, CINAR O, EVREN B, ULKU M, KARABULUT V, UMUT G (2013). The mediator roles of trait anxiety, hostility, and impulsivity in the association between childhood trauma and dissociation in male substance-dependent inpatients. Compr Psychiatry.

[48] DOTY B. The construct of resilience and its application to the context of political violence. Pursuit 2010, 1:11.

[49] DAVYDOV DM, STEWART R, RITCHIE K, CHAUDIEU I. Resilience and mental health. Clinical psychology review. 2010 July, 30. 479–95. 10.1016/j.cpr.2010.03.003. 10.1016/j.cpr.2010.03.003 20395025

[50] ROBERTS B, BROWNE J (2011). A systematic review of factors influencing the psychological health of conflict-affected populations in low and middle-income countries. Glob Public Health.

[51] OZBAY F, JOHNSON DC, DIMOULAS E, MORGAN CA, CHARNEY D, SOUTHWICK S (2007). Social support and resilience to stress: from neurobiology to clinical practice. Psychiatry (Edgmont).

[52] SMITH RE, SMOLL FL, PTACEK JT (1990). Conjunctive moderator variables in vulnerability and resiliency research: life stress, social support and coping skills, and adolescent sport injuries. J Pers Soc Psychol.

[53] SOUTHWICK SM, SIPPEL L, KRYSTAL J, CHARNEY D, MAYES L, PIETRZAK R (2016). Why are some individuals more resilient than others: the role of social support. World Psychiatry.

[54] ARAGONA M, PUCCI D, MAZZETTI M, MAISANO B, GERACI S (2013). Traumatic events, post-migration living difficulties and post-traumatic symptoms in first generation immigrants: a primary care study. Ann Ist Super Sanita.

[55] PIGNON B, AMAD A, PELISSOLO A, FOVET T, THOMAS P, VAIVA G, ROELANDT JL, BENRADIA I, ROLLAND B, GEOFFROY PA. Increased prevalence of anxiety disorders in third-generation migrants in comparison to natives and to first-generation migrants. J Psychiatr Res. 2018, 20;102:38–43. [Epub ahead of print]. 10.1016/j.jpsychires.2018.03.007 29597072

[56] FORTUNA LR, PORCHE MV, ALEGRIA M (2008). Political violence, psychosocial trauma, and the context of mental health services use among immigrant Latinos in the United States. Ethn Health.

[57] DOBSON KS, DOZOIS DJA (2008). Risk factors in depression.

[58] KALTMAN S, GREEN BL, METE M, SHARA N, MIRANDA J (2010). Trauma, depression, and comorbid PTSD/depression in a community sample of Latina immigrants. Psychol Trauma.

[59] GNANAVEL S, ROBERT RS. Diagnostic and Statistical Manual of Mental Disorders, Fifth Edition, and the Impact of Events Scale-Revised. Chest. 2013;144(6). 10.1378/chest.13-1691 24297138

[60] VENTRIGLIO A, BHUGRA D. Migration, trauma and resilience. In: Trauma and migration: Cultural factors in the diagnosis and treatment of traumatised immigrants. Springer, New York, 2015.

[61] LEBRUN A, HASSAN G, BOIVIN M, FRASER SL, DUFOUR S, LAVERGNE C. Review of child maltreatment in immigrant and refugee families. Can J Public Health. 2016 Mar 14, 106(7 Suppl 2):eS45–56. doi: 10.17269/cjph.106.4838. 10.17269/CJPH.106.4838 PMC6972055 26978697

[62] VAUGHN MG, SALAS-WRIGHT CP, HUANG J, QIAN Z, TERZIS LD, HELTON JJ (2017). Adverse childhood experiences among immigrants to the United States. J Interpers Violence.

[63] TAILLIEU TL, BROWNRIDGE DA, SAREEN J, AFIFI TO. Childhood emotional maltreatment and mental disorders: results from a nationally representative adult sample from the United States. Child Abuse Negl 2016, 59:1–12. doi: 10.1016/j.chiabu.2016.07.005. Epub 2016 Aug 1. PubMed PMID: 27490515. 10.1016/j.chiabu.2016.07.005 27490515

[64] NORMAN RE, BYAMBAA M, De R, Butchart A, Scott J, Vos T (2012). The long-term health consequences of child physical abuse, emotional abuse, and neglect: a systematic review and meta-analysis. PLoS Med.

[65] MOLNAR BE, BERKMAN LF, BUKA SL (2001). Psychopathology, childhood sexual abuse and other childhood adversities: relative links to subsequent suicidal behaviour in the US. Psychol Med.

[66] ROY A, JANAL M (2006). Gender in suicide attempt rates and childhood sexual abuse rates: is there an interaction?. Suicide Life Threat Behav.

[67] SARCHIAPONE M (2009). Childhood trauma as a correlative factor of suicidal behavior – via aggression traits. Similar results in an Italian and in a French sample. Eur Psychiatry..

[68] MENON V, SARKAR S, KATTIMANI S, MATHAN K (2015). Do personality traits such as impulsivity and hostility-aggressiveness predict severity of intent in attempted suicide? Findings from a record based study in South India. Indian J Psychol Med.

